# Comment on “Gender-Based Differences and Barriers in Skin Protection Behaviors in Melanoma Survivors”

**DOI:** 10.1155/2016/1345726

**Published:** 2016-11-16

**Authors:** Vinayak K. Nahar, Amanda K. Hutcheson, Javier F. Boyas, Stephanie K. Jacks, Robert T. Brodell

**Affiliations:** ^1^Department of Dermatology, University of Mississippi Medical Center, Jackson, MS, USA; ^2^Department of Health, Physical Education, and Exercise Science, School of Allied Health Sciences, Lincoln Memorial University, Harrogate, TN, USA; ^3^Department of Health Science, College of Human Environmental Sciences, The University of Alabama, Tuscaloosa, AL, USA; ^4^Department of Social Work, School of Applied Sciences, University of Mississippi, University, MS, USA

In a recent published article in this journal, Chen and colleagues reported gender-based differences in skin protection behaviors among melanoma survivors [1]. This is a significant contribution to the growing literature in the area of skin cancer prevention among population groups at high risk for the development of future skin cancers. We would like to commend the authors for their research and build on their contributions by putting forth some practical implications.

According to Chen et al., a vast majority (87%) of melanoma survivors conducted skin self-examination (SSE) for abnormal markings more often, with females more likely to conduct these exams than males. Overall, this behavior change is encouraging; however, it is possible that these survivors are not thoroughly checking all body areas [2]. A study with 229 melanoma survivors at a large comprehensive cancer center in the Northeastern United States demonstrated that only 13.7% performed a thorough SSE [3]. More recently, a study involving 176 melanoma survivors indicated that only 14.2% had checked all areas of their body in the past two months [4]. One of the most common reasons given by melanoma survivors for not performing SSE in the past two months was, “I do not know what to look for.” Moreover, it was found that knowledge of the ABCDE rule among melanoma survivors was dismally low. This could be improved by reviewing ABCDE handouts during office visits or sending survivors follow-up email reminders that contain the ABCDE criteria. These reminders should include educational information, and practitioners could even consider testing patients' knowledge to determine if they understood the material they were presented.

The findings from Chen et al.'s study have implications for future programs to improve sun safety behavior among melanoma survivors. A greater emphasis should be placed on recognizing that gender differences exist in terms of using sun protective behaviors. When developing preventive and intervention programs, dermatologists and health promotion practitioners have to determine the sun-safe behaviors used by each group. It is clear from this research that both sexes do employ multiple sun protective methods. However, these behaviors may be altered by lifestyle differences between men and women. For example, as stated by the authors, men may wear more wide-brim hats because they may be employed in outdoor work. This may partially explain why men, compared to women, also reported using less sunscreen, were less able to seek shade, and/or were less likely to limit outdoor activity. Outdoor workers may not have the ability to stop work to use sun protective measures. Thus, prevention and intervention programs also must pay close attention to gender differences with respect to occupational hazards. Failing to do so may elevate the risk of recurrence for this already vulnerable population.

Women in this study used more preventive sun safety measures compared to men. In previous studies, women were more likely to exhibit positive attitudes towards sun protection behavior, report fewer barriers to sun protection behavior, and view skin damage from sun exposure as more severe because of its cosmetic effects (wrinkles, brown spots, yellowing, and sagging of sun-exposed skin). These reasons provide a partial explanation for why women, relative to men, engaged in more preventive behaviors. With regard to men, previous research shows that they are more likely to forget to engage in sun protection when compared to women [5]. Practitioners and healthcare providers should address these gender differences in sun protection behaviors in the development of health promotion interventions, such as through the use of reminders and provisions of personal protection equipment and the use of tailored intervention components to address the differences in attitude and barriers to sun protection reported in previous research.

Another important result that emerged from Chen et al.'s study showed that the majority (60%) of the melanoma survivors reported using SPF 30 sunscreen less often than prior to their diagnosis. Dermatologists and health promotion practitioners should address the lack of sunscreen use and modify beliefs regarding sunscreen use following melanoma diagnosis in prevention efforts among melanoma survivors. Healthcare practitioners should engage in patient counseling that not only encourages sunscreen use but also instructs patients on the proper use and frequent reapplication of sunscreen to protect against sunburns [6]. Patient counseling should also emphasize the common perceptions regarding sunscreen use discussed by Chen and colleagues. In addition to counseling, health professionals may consider the provision of personal protection equipment, including sunscreen, which has been shown to be efficacious in increasing sunscreen use [7]. Further, when developing interventions to address sunscreen use among melanoma survivors, health promotion practitioners may consider developing tailored, theoretically grounded interventions addressing barriers, benefits, and attitudes regarding sunscreen use specific to melanoma survivors [8]. Previous research suggests that tailored interventions addressing known theoretical constructs may be more efficacious in increasing sunscreen use than general intervention materials [8].

In closing, based on Chen et al.'s findings, gender differences in skin protection practices after melanoma treatment do exist. However, the reasons for those differences are not yet clear. Perhaps future research should use qualitative methods to provide a more in-depth understanding of why preventative practices vary between male and female melanoma survivors. It may be that prevention and intervention programs have to be adapted to account for gender differences in the use of sun-safe practices.

## References

[1] Chen J., Shih J., Tran A. (2016). Gender-based differences and barriers in skin protection behaviors in melanoma survivors. *Journal of Skin Cancer*.

[2] Nahar V. K., Ford M. A., Brodell R. T. (2016). Skin cancer prevention practices among malignant melanoma survivors: a systematic review. *Journal of Cancer Research and Clinical Oncology*.

[3] Manne S., Lessin S. (2006). Prevalence and correlates of sun protection and skin self-examination practices among cutaneous malignant melanoma survivors. *Journal of Behavioral Medicine*.

[4] Coups E. J., Manne S. L., Stapleton J. L., Tatum K. L., Goydos J. S. (2016). Skin self-examination behaviors among individuals diagnosed with melanoma. *Melanoma Research*.

[5] Kasparian N. A., McLoone J. K., Meiser B. (2009). Skin cancer-related prevention and screening behaviors: a review of the literature. *Journal of Behavioral Medicine*.

[6] Planta M. B. (2011). Sunscreen and melanoma: is our prevention message correct?. *The Journal of the American Board of Family Medicine*.

[7] Horsham C., Auster J., Sendall M. C. (2014). Interventions to decrease skin cancer risk in outdoor workers: update to a 2007 systematic review. *BMC Research Notes*.

[8] Manne S., Jacobsen P. B., Ming M. E., Winkel G., Dessureault S., Lessin S. R. (2010). Tailored versus generic interventions for skin cancer risk reduction for family members of melanoma patients. *Health Psychology*.

